# Increased sensitivity to SMAC mimetic LCL161 identified by longitudinal ex vivo pharmacogenomics of recurrent, *KRAS* mutated rectal cancer liver metastases

**DOI:** 10.1186/s12967-021-03062-3

**Published:** 2021-09-08

**Authors:** Kushtrim Kryeziu, Seyed H. Moosavi, Christian H. Bergsland, Marianne G. Guren, Peter W. Eide, Max Z. Totland, Kristoffer Lassen, Andreas Abildgaard, Arild Nesbakken, Anita Sveen, Ragnhild A. Lothe

**Affiliations:** 1 Department of Molecular Oncology, Institute for Cancer Research, Oslo University Hospital, Nydalen, P. O. Box 4953, 0424 Oslo, Norway; 2 Institute for Clinical Medicine, Faculty of Medicine, University of Oslo, Oslo, Norway; 3 Department of Oncology, Oslo University Hospital, Oslo, Norway; 4 Department of Hepato-Pancreato-Biliary Surgery, Oslo University Hospital, Oslo, Norway; 5 Institute of Clinical Medicine, University of Tromsø, Tromsø, Norway; 6 Division of Radiology and Nuclear Medicine, Oslo University Hospital, Oslo, Norway; 7 Department of Gastrointestinal Surgery, Oslo University Hospital, Oslo, Norway

**Keywords:** Rectal cancer, Liver metastasis, Patient-derived organoid, LCL161

## Abstract

Tumor heterogeneity is a primary cause of treatment failure. However, changes in drug sensitivity over time are not well mapped in cancer. Patient-derived organoids (PDOs) may predict clinical drug responses ex vivo and offer an opportunity to evaluate novel treatment strategies in a personalized fashion. Here we have evaluated spatio-temporal functional and molecular dynamics of five PDO models established after hepatic re-resections and neoadjuvant combination chemotherapies in a patient with microsatellite stable and *KRAS* mutated metastatic rectal cancer. Histopathological differentiation phenotypes of the PDOs corresponded with the liver metastases, and ex vivo drug sensitivities generally reflected clinical responses and selection pressure, assessed in comparison to a reference data set of PDOs from metastatic colorectal cancers. PDOs from the initial versus the two recurrent metastatic settings showed heterogeneous cell morphologies, protein marker expression, and drug sensitivities. Exploratory analyses of a drug screen library of 33 investigational anticancer agents showed the strongest ex vivo sensitivity to the SMAC mimetic LCL161 in PDOs of recurrent disease compared to those of the initial metastasis. Functional analyses confirmed target inhibition and apoptosis induction in the LCL161 sensitive PDOs from the recurrent metastases. Gene expression analyses indicated an association between LCL161 sensitivity and tumor necrosis factor alpha signaling and *RIPK1* gene expression. In conclusion, LCL161 was identified as a possible experimental therapy of a metastatic rectal cancer that relapsed after hepatic resection and standard systemic treatment.

## Introduction

“Living biobanks” of patient-derived tumor organoids (PDOs) can model histopathological and pharmacogenomic heterogeneity in cancer [1–5]. Co-clinical studies have shown that PDOs can predict clinical responses to both targeted agents and chemotherapies in an observational setting [6, 7]. However, tumor heterogeneity is a major driving force of treatment failure [8]. PDOs established from distinct liver lesions of patients with metastatic colorectal cancer (CRC) have shown that inter-metastatic heterogeneity of drug sensitivities is limited [9], but systemic anticancer treatment inflicts selective pressures that can drive the evolution of drug-resistant subclones [10–13]. Longitudinal monitoring may therefore guide treatment adaptation, as illustrated by rechallenge with anti-EGFR therapy against metastatic CRC guided by monitoring of *KRAS* mutation levels in the blood [14, 15]. However, most studies of cancer cell drug vulnerabilities and functional modeling are cross-sectional and provide 'snapshots' of a single point in time [1, 2, 7, 16].

Patients diagnosed with *KRAS* mutated CRC have limited systemic treatment options when resistance to standard combination chemotherapies with or without antiangiogenic agents occurs [17, 18]. The mechanisms of chemoresistance in KRAS-driven cancers are not well understood. However, acquired chemoresistance might be associated with evasion of apoptosis via dysregulated expression of inhibitor of apoptosis proteins (IAPs) [19, 20]. IAPs can directly inhibit caspases or divert death-inducing signals of the tumor necrosis factor (TNF) pathway into prosurvival signals via activation of the proliferative transcriptional programs of Receptor-interacting protein kinase 1 (RIPK1) and Nuclear factor kappa-B (NF-κB) [19, 21–23]. Endogenous regulation of IAPs is mediated by second mitochondria-derived activator of caspases (SMACs), which are released from the mitochondria into the cytosol in response to apoptotic stimuli, and thus free the caspases to execute pro-apoptotic functions. Small therapeutic compounds mimicking SMACs can promote apoptosis in cancers with deregulated IAP expression [19]. However, the clinical success of SMAC mimetics as anticancer agents has been limited by the lack of predictive biomarkers [24].

Here, we report a longitudinal, observational co-clinical study of standard combination chemotherapies in a patient with recurrent, KRAS mutated liver metastases from rectal cancer. A SMAC mimetic was identified as a novel therapeutic opportunity in PDOs of the recurrent tumors.

## Materials and methods

### Patient and samples

The patient diagnosed with metastatic rectal cancer was surgically treated for liver metastases at Oslo University Hospital on three occasions, between December 2017 and February 2020. Parallel samples from resected specimens were fresh frozen (− 80 °C) for molecular profiling, or transported ice-cold in basal media (Advanced DMEM/F-12 supplemented with 10 mM HEPES, 2 mM GlutaMAX all from Thermo Fischer Scientific and 100 µg/ml Primocin from Invivogen) for organoid cell culturing within 24 h. A sample set of similarly processed liver metastases (n = 46) from 23 patients diagnosed with metastatic CRC described in ref [9] have been used as the reference dataset in this study.

### Culturing patient-derived tumor organoids

Samples from resected tumor specimens (2.5—6 × 7 mm in size) were minced into 0.1- 0.5 mm fragments, washed with ice-cold basal culture media, strained with a 70 µm pore mesh, and collected by centrifugation at 400*g* 4 °C for 5 min. The pellet was suspended in Growth Factor Reduced Matrigel (Corning), dispensed onto pre-warmed 6-well tissue culture plates as 25 µl drops, overlaid with 3 ml organoid growth media supplemented with 10 µM Y-27632 and then incubated at 37 °C in a humidified 5% CO_2_ atmosphere. Organoid growth media consisted of basal culture media supplemented with 1xB27 supplement (Gibco), 10 nM [Leu15]-Gastrin I (Sigma) and 1 mM N-acetyl-l-cysteine (Sigma) and the following niche factors: 50 ng/mL for EGF (Gibco), 100 ng/mL for Noggin (Preprotech), 500 nM for TGF-β receptor type I inhibitor A83-01 (Tocris) and 10 µM for p38 MAP kinase inhibitor SB202190 (Sigma). Organoid growth media without Y-27632 was refreshed every two to four days. Organoids were passaged by digestion with TrypLE Express (Gibco) for 5 min at 37 °C supplemented with 10 µM Y-27632. Contamination-free organoid cultures were ensured using MycoAlert Mycoplasma Detection Assay (Lonza) within a week after the functional assays or before cryopreservation. The authenticity of the cultures was verified by comparison to the respective tumor tissues using AmpFLSTR Identifiler PCR Amplification Kit (Thermo Fisher Scientific).

### Drug sensitivity screening

A medium throughput drug screen of a custom library of 33 clinically relevant small molecule inhibitors in eight different concentrations each, and three drug combinations in seven concentrations was performed as previously described [9]. The drug library was selected for clinical relevance in CRC, and included all small molecules approved to treat CRC, drugs with emerging clinical evidence of activity against CRC, and selected drugs that are either approved or in clinical testing for other cancer types. Selection among agents with the same mechanism of action was based on robust drug sensitivity scores tested in CRC cell lines [25] and the furthest development in clinical studies. The setup of one treatment reaction included 40 fold drug concentration (preprinted with liquid acoustic dispensing technology Echo 550, Labcyte Inc. at the High Throughput Biomedicine Unit at the Institute for Molecular Medicine Finland) overlaid with 10 µl of 50% Matrigel, followed by a suspension of 450–600 prestrained organoids (70 µm mesh size) with 30 µl of 3% Matrigel. Two parallel replicas per sample were incubated with the drugs and positive (100 µM benzethonium chloride, n = 9 wells) and negative (0.1% DMSO, n = 13 wells) controls for 96 h at 37 °C in a humidified 5% CO_2_ atmosphere, and analyzed by the CellTiter-Glo 3D Cell Viability Assay (Promega) according to the manufacturer's instructions, prior to luminescence measurement with a Victor 3 microplate reader (Perkin Elmer). Luminescence readouts were rescaled to relative viability based on the median of the negative and positive control wells per plate (separately for the two technical replicates). Data from technical replicates were combined to estimate dose–response curves [9]. Drug sensitivity scores were calculated as described in Yadav et al. (2014) [26].

### Growth rate adjustment of drug activities

For growth rate adjusted drug sensitivity scores (_GR_DSS), area under the curve was calculated using growth rate in place of relative viability based on a previously developed method for estimation of growth fold-changes [27]. To estimate growth rate, the following steps were performed. Micrographs were captured at baseline seeding of each drug screen, as well as after four days of incubation with the negative and positive controls using an EVOS FL microscope (Thermo Fisher Scientific). The volume of more than 150 structures of each sample at baseline, and of 150 structures after 96 h incubation with the negative control, were measured manually using ImageJ (Fiji) based on the longest (l) and shortest (s) diameters of the structures using the following formula:

$$V = \frac{{\uppi l{s^2}}}{6}$$

Volume fold-changes ($\Delta \mathrm{v}$) were calculated as the mean volume of structures after incubation with negative control divided by the volume of the baseline structures, and used to calculate organoid doubling time (*Td*) as follows:

$$\Delta \mathrm{v}={2}^{Te/Td}\to Td=\frac{Te \mathrm{log}(2)}{\mathrm{log}(\Delta v)}=\frac{Te}{{log}_{2}(\Delta v)}$$

where *Te* is the experimental duration (typically 96 h).

Drug screen quality was evaluated based on the strictly standardized mean difference (SSMD) metric of the raw luminescence readouts from DMSO treated (neg) and benzethonium chloride treated (pos) conditions as follows:

$$SSMD=\frac{mean\left(pos\right)-mean(neg)}{\sqrt{var(pos)+var\left(neg\right)}}$$

Samples with SSMD < 3 were discarded and repeated. For the included screens, the median SSMD was 9.3 [range: 3.5–34].

### DNA/RNA extractions and mutation analyses

DNA and RNA were extracted from fresh-frozen tissue samples and PDOs using the Allprep DNA/RNA/miRNA Universal kit (Qiagen GmBH, Hilden, Germany), following the manufacturer’s instructions. Microsatellite instability (MSI) status and *KRAS, NRAS, BRAF* hotspot mutation status were determined as previously described [9, 28].

### Gene expression analyses

Gene expression profiles were generated for all resected liver metastasis tissue samples and their corresponding PDOs using the GeneChip Human Transcriptome Array 2.0 (HTA 2.0) with 100 ng of total RNA as input and following the manufacturer's protocol (Thermo Fisher Scientific). Raw intensity data were pre-processed, normalized and log2 transformed for tissue samples and PDOs separately by robust multi-array average (RMA) method implemented in justRMA function in the R package affy [29] using the custom Entrez CDF file (v24) from Brainarray [30]. Entrez IDs were converted to HGNC gene symbols using the org.Hs.eg.db package (v 3.7.0) from Bioconductor. Single-sample gene set enrichment analyses of gene signatures of response and resistance to 5-fluorouracil (5-FU) [31], as well as the “hallmark” gene sets (n = 50; retrieved from the Molecular Signatures Database, v7.0 [32]) were performed by the gsva function (gene set variation analysis) in the R package GSVA [33]. Sensitivity to LCL161 was also analyzed in relation to log2 expression signals of previously suggested target genes encoding mediators of the TNF alpha pathway and apoptosis regulators [34].

### Immunostaining

All PDO lines and one liver metastasis tissue were formalin-fixed, paraffin-embedded and assembled in a microarray with 4 mm cores, sliced at 3 µm sections, and stained for hematoxylin and eosin. A section was also stained and analyzed for caudal type homeobox 2 (CDX2), cytokeratin 20 (CK20), E-cadherin (ECAD) and cytokeratin 7 (CK7) using multiplexed fluorescence staining based on Opal kits and reagents (product numbers NEL810001KT and FP1495001KT, Akoya Biosciences) and multispectral imaging (Vectra3 imaging system, Akoya Biosciences). The following antibodies and fluorophores were used to detect each target: anti-CDX2 (1:400, clone EPR2764Y, Cell Marque) detected by Opal 570, anti-CK20 (1:1000, clone Ks20.8, Agilent Dako) detected by Opal 520, anti-ECAD (1:10.000, clone 36, BD Biosciences) detected by Opal 690, anti-CK7 (1:400, clone OV-TL 12/30, Agilent Dako) detected by Opal 620. Cell nuclei were stained with DAPI. Multispectral images were unmixed in Inform Software (Akoya Biosciences) and all images displayed are scaled equally.

### Western Blot analyses

7.5 × 10^3^ PDOs were incubated for 48 h with 200 µM LCL161 or 0.01% DMSO. After drug incubation, the cells were washed with 1xPBS, scraped, collected, lysed in 100 µl SDS electrophoresis sample buffer (10 mM Tris (pH 6.8), 15% w/v glycerol, 3% w/v SDS, 0.01% w/v bromphenol blue, and 5% v/v 2-mercaptoethanol), sonicated and heated at 95 °C for 5 min. Protein concentration was determined using the RC-DC Protein Assay kit (Bio-Rad) according to the manufacturer's recommendation. Protein lysates were separated on 12% SDS-PAGE gels by electrophoresis then blotted onto PVDF membranes using a Trans-Blot® Turbo™ Transfer System (Bio-Rad). The primary antibodies used were: c-IAP1 (D5G9) Rabbit mAb #7065, c-IAP2 (58C7) Rabbit mAb #3130, XIAP (3B6) Rabbit mAb #2045, Survivin (71G4B7) Rabbit mAb #2808, Cleaved Caspase-8 (Asp384) (6B6) Mouse mAb #9747, Caspase-7 (C7) Mouse mAb #9494, Caspase-3 Antibody #9662, PARP Antibody Rabbit #9542, Cleaved PARP (Asp214) (D64E10) XP(R) Rabbit mAb #5625 all from Cell Signaling at 1:1000 dilution as well as anti-β-actin #A2228 from Sigma-Aldrich at 1:5000 dilution. HRP conjugated secondary antibodies (Bio-Rad) were used at 1:5000 dilution and chemiluminescence detected using SuperSignal West Dura Extended Duration Substrate kit (Thermo Fisher Scientific) on a Bio-Rad image station. Protein band intensities were quantified with Image Lab V5.2 Software from Bio-Rad.

## Results

### Case presentation and PDO establishment

**Fig. 1:**
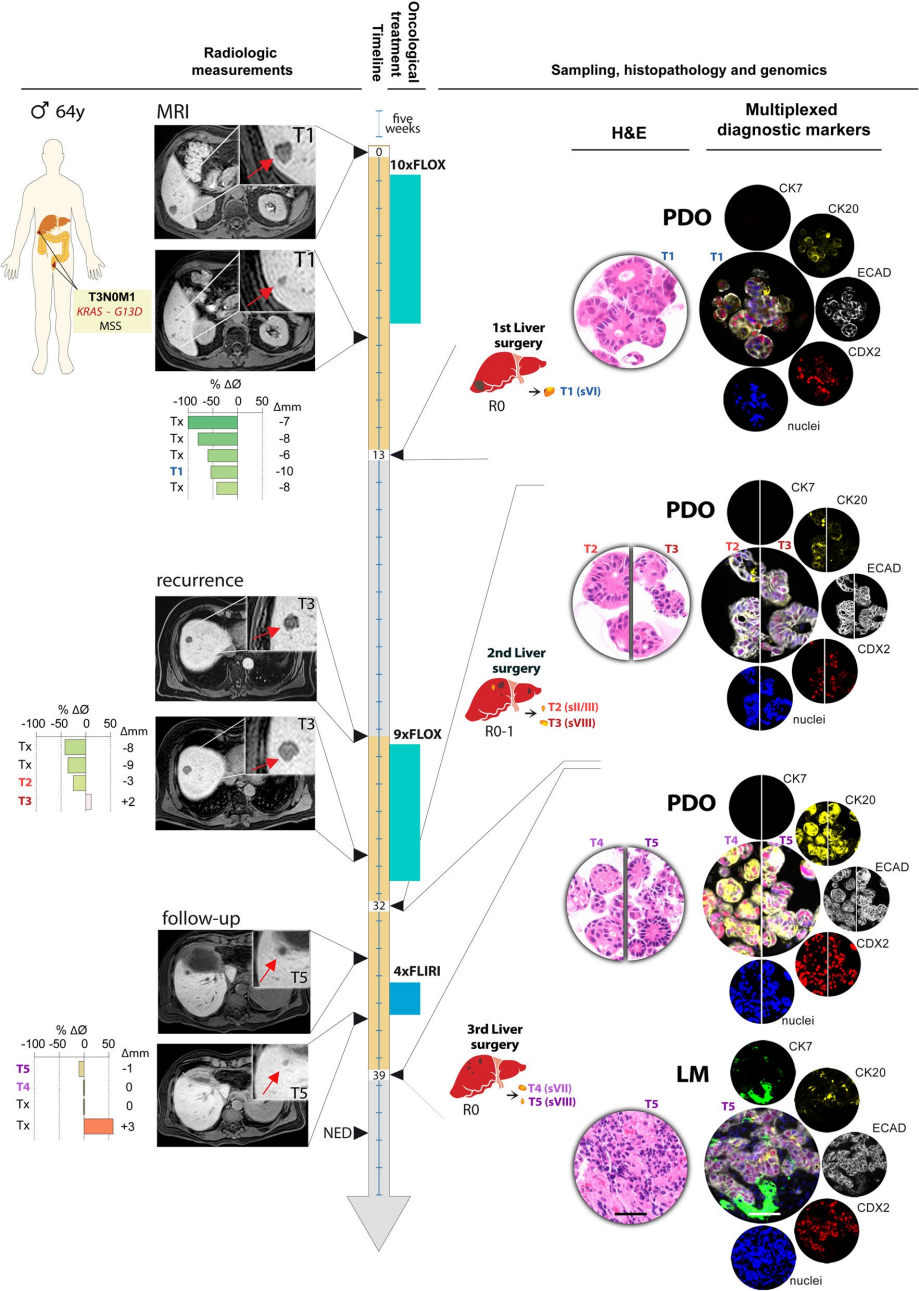
Clinical history of the patient. One increment in the timeline ruler corresponds to five weeks, and numbers indicate months starting from the MRI before neoadjuvant systemic treatment to surgical interventions. Left column shows MRIs (transverse plane) before and after neoadjuvant combination chemotherapy for all three surgeries, and bar graphs indicate radiologic response measurements of individual lesions (T1–T5 are sampled lesions, Tx are non-sampled lesions) as the percent difference in diameter (% ΔØ) and as the absolute size difference in mm (Δmm). Columns to the right indicate (from left) chemotherapy (number of cycles and duration), resection of liver metastases, tissue sampling from the indicated liver segment (Roman numeral), immunohistochemistry of diagnostic markers in PDOs and one tissue sample. Scale bar = 50 µm. *FLOX* 5-fluorouracil, leucovorin, and oxaliplatin, *FLIRI* 5-fluorouracil, leucovorin, and irinotecan, *PDO* patient-derived organoid, *LM* liver metastasis, *NED* No evidence of disease, *H&E* hematoxylin and eosin

A 64 year old man was diagnosed with *KRAS* mutated (codon G13D), microsatellite stable rectal cancer disseminated to the liver (T3N0M1; Fig. 1). The patient was scheduled for neoadjuvant combination chemotherapy with 5-FU, leucovorin and oxaliplatin (FLOX) followed by liver resection, and the metastases showed partial response (according to response evaluation criteria in solid tumors 1.1) [35], with a mean shrinkage of the measured tumor deposits of 75% (n = 5, range: 45%–100%) (Fig. 1 upper left). One lesion was resected (T1: 10 mm diameter reduction, corresponding to 55% tumor shrinkage; clear resection margin) and submitted for organoid culturing and genomics [9]. All other lesions were treated with microwave ablation. Histopathological hematoxylin and eosin stains of the PDOs showed three-dimensional cell structures with well- and poorly-defined epithelial layers and small- or absent lumens, indicating a moderately differentiated phenotype (Fig. 1 upper right). ECAD, CDX2, and CK20 were expressed, whereas CK7 was absent, supporting epithelial CRC tissue origin.

The patient remained disease-free until ten metastases were detected at new locations in the liver by magnetic resonance imaging (MRI) at follow-up 11.7 months after surgery. The patient was scheduled for chemotherapy followed by hepatic re-resection, and the recurrent lesions showed partial response to neoadjuvant FLOX (Fig. 1 middle left). All lesions were resected with clear margins, and the two lesions with poorest response to chemotherapy remained amenable to sampling, organoid culturing and genomics (T2: 3 mm diameter reduction, corresponding to 25% tumor shrinkage; T3: 2 mm diameter increase, 11% tumor growth). Both PDOs showed a more undifferentiated phenotype compared to the PDO from the first resection, with small or absent lumens, and disorganized epithelial cell layers with ECAD, CDX2 and CK20 expression (Fig. 1 middle right).

Ten weeks after re-resection, one previously undetected metastatic lesion and three new lesions were seen on follow-up MRI. Four cycles of neoadjuvant treatment with 5-FU, leucovorin and irinotecan (FLIRI) resulted in heterogeneous responses among the lesions (Fig. 1 bottom left). Two of the new lesions were resected and submitted for organoid culturing and genomics (T4: no diameter change of the 6 mm large tumor; T5: 1 mm diameter reduction corresponding to 9% tumor shrinkage), and the two other lesions were treated with radiofrequency ablation. Similar to PDOs from the second liver surgery (T2 and T3), PDOs from T4 and T5 exhibited an undifferentiated morphology, but with a slightly stronger CK20 and CDX2 expression (Fig. 1 bottom right). For reference, the corresponding tissue sample from the T5 lesion was stained for the same diagnostic markers, showing that the PDO retained the undifferentiated phenotype and expression patterns of the tumor.

### Spatio-temporal co-clinical evaluation of standard chemotherapies

**Fig. 2:**
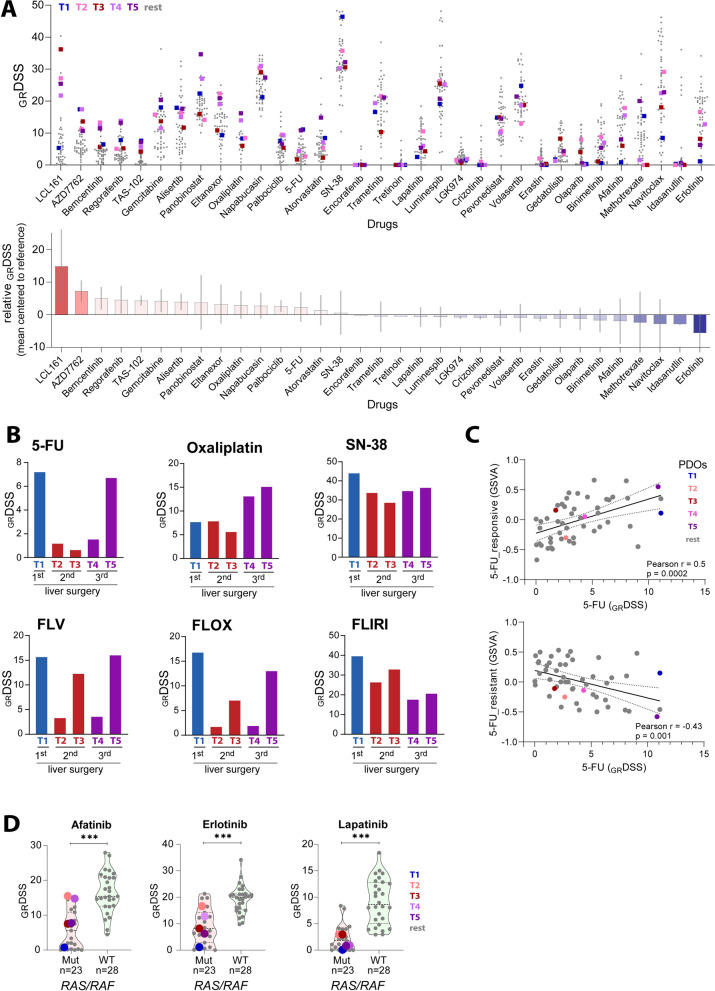
Drug sensitivity analyses of a medium throughput drug library. (**A**, upper panel) _GR_DSS of 33 drugs in T1–T5 (colored dots) as compared to the reference PDO lines (grey dots, n = 46). (**A**, lower panel) Mean _GR_DSS of 33 drugs in T1–T5 centered to the mean of the entire dataset (n = 51). The grey line within the bars indicates standard deviation of the respective drug’s _GR_DSS for T1–T5 lesions. **B** Drug activities of 5-FU, oxaliplatin, SN-38 (active metabolite of irinotecan) as single agents and combination therapies in T1-T5 PDOs. **C** Scatter plots of 5-FU drug sensitivity scores (growth rate adjusted) and GSVA scores of signatures of 5-FU sensitivity and resistance in PDOs from this patient (colored as indicated) and a reference PDO dataset (grey). **D** EGFR inhibitor activities and their association with *RAS/RAF* mutation status analyzed in 23 mutated and 28 wild type PDOs. _GR_DSS—Growth adjusted drug sensitivity scores, 5-FU 5-fluorouracil, FLV 5-FU + 10 μM leucovorin, FLOX 5-FU and oxaliplatin at 1:1 ratios + 10 μM leucovorin, FLIRI 5-FU and SN-38 at 100:1 ratios + 10 μM leucovorin, GSVA—gene set variation analysis

All PDOs were screened with a customized medium-throughput drug library incorporating 33 single agents and three 5-FU-based drug combinations with Leucovorin (FLV), Oxaliplatin (FLOX), and SN-38 (FLIRI) (Fig. 2A, B). Co-clinical evaluation of FLOX showed that the PDO from the first resection reflected the strong clinical response in the corresponding T1 lesion (Fig. 1 upper left and Fig. 2B). The _GR_DSS in the T1 PDO indicated a particularly strong sensitivity to 5-FU, also in comparison to our reference dataset of 46 PDOs from 23 patients with resected CRC liver metastases [9] (Fig. 2A), suggesting that the clinical response was primarily driven by this agent. Sensitivity to 5-FU was much lower in the two PDOs from the second resection, and sensitivity to oxaliplatin was largely unchanged, consistent with the weaker clinical responses to FLOX in the corresponding lesions (T2 and T3). Notably, heterogeneous sensitivity to combination therapies with FLV and FLOX between T2 and T3 PDOs did not correspond to the relative radiological responses of the corresponding lesions (Fig. 2B), potentially related to the poor clinical response of both lesions. T4 and T5 PDOs from the third resection showed higher sensitivity to oxaliplatin and lower sensitivity to FLIRI than PDOs from previous resections, possibly associated with a different selection pressure after the change in treatment from FLOX to FLIRI in the third neoadjuvant setting. 5-FU showed heterogeneous activity between T4 and T5 PDOs, not consistent with the poor clinical responses to FLIRI in corresponding lesions, or the small difference in ex vivo sensitivity to FLIRI between the PDOs.

Across all PDOs and the reference PDO dataset, gene expression signatures of response and resistance to 5-FU [31], analyzed as GSVA scores, were significantly correlated to the measured 5-FU sensitivity (Fig. 2C).

### Increased sensitivity to SMAC mimetic LCL161 in recurrent lesions

**Fig. 3:**
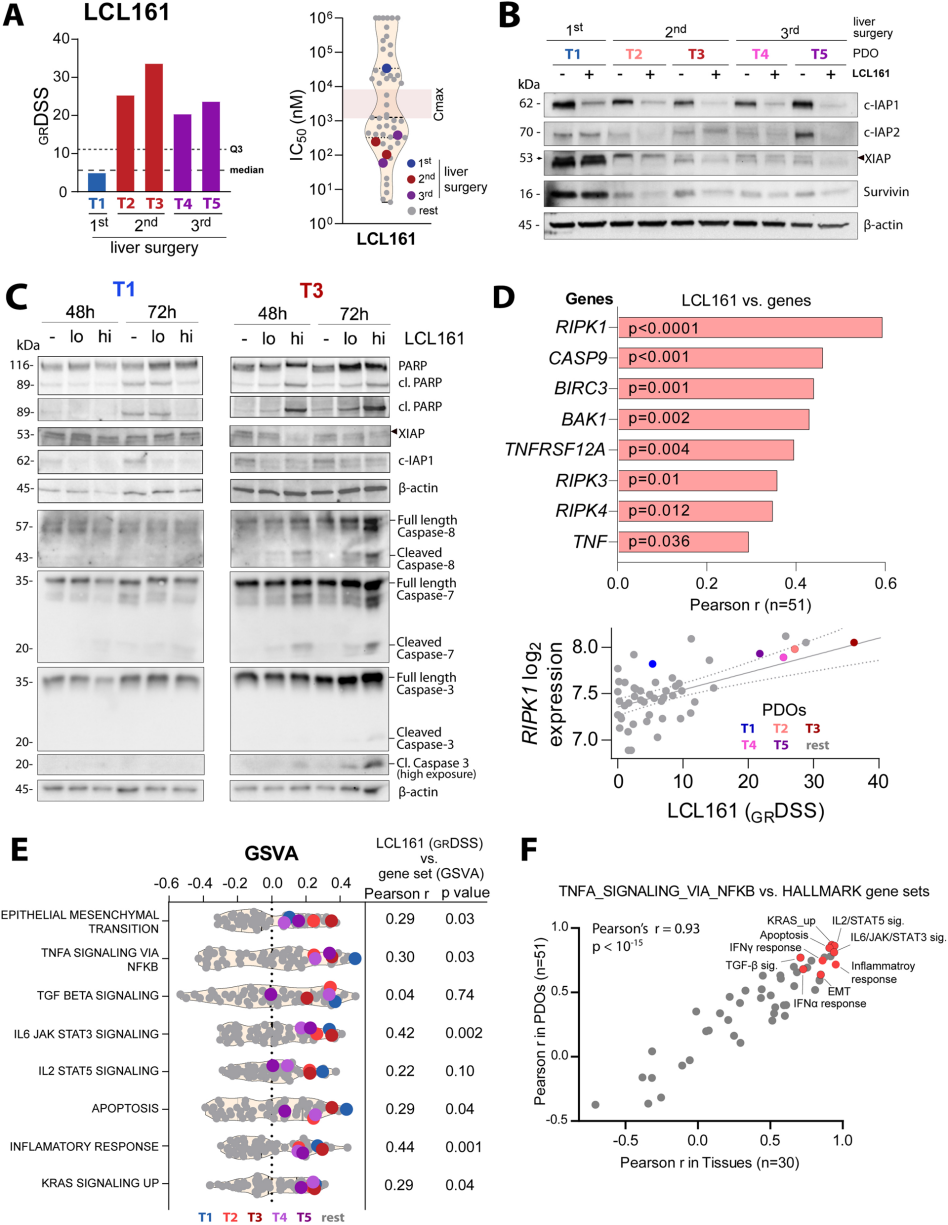
Anticancer activities of LCL161. **A** LCL161 _GR_DSS and IC_50_ distribution in T1-T5 PDOs. Reference PDOs are indicated in grey. **B** Protein expression of IAPs analyzed with Western blotting after treatment with DMSO (−) and 200 nM LCL161 ( +) for 48 h. **C** Time dependent effects of 200 nM (lo) and 2000 nM (hi) LCL161 on PARP cleavage, inhibition of XIAP and c-IAP1, Caspase- 8, 7 and 3 cleavage in LCL161-resistant T1 and sensitive T3 PDOs. (**D**, upper panel) Pearson correlation of significantly associating genes of RIP kinase family, gene members of the TNF signaling and other apoptosis regulators with _GR_DSS of LCL161. (**D**, lower panel) Scatter plot of *RIPK1* gene expression versus LCL161 drug activity in PDOs. **E**. Enriched “Hallmark” gene sets in T1–T5 PDOs (colored) as compared to the reference PDO lines n = 46 (grey). **F** Scatter plot of Pearson’s correlation coefficients (r) between the “TNFA signaling via NFKB” gene set and remaining 49 “Hallmark” gene sets analyzed in 51 PDO lineages (vertical axis) and 30 liver metastasis tissue samples (horizontal axis). Highlighted in red are the gene sets that are enriched in T1-T5 PDOs and significantly associated with LCL161 drug activity. _*GR*_*DSS* Growth adjusted drug sensitivity scores, *IC*_*50*_ the half maximal inhibitory concentration, *C*_*max*_ the maximum serum concentration of a drug, *kDa* kilodalton, *GSVA* gene set variation analysis

Low sensitivity to EGFR inhibitors was confirmed in these *KRAS* mutated PDOs, both relative to other anti-cancer agents and relative to *RAS* wild-type PDOs in the reference dataset (Fig. 2A and D). The highest mean sensitivity score in the five PDOs across the complete tested panel of single agents was found for the SMAC mimetic LCL161 (Fig. 2A). However, most of the drugs showed intra-patient heterogeneity and differential activities among the five PDOs. Largest difference in sensitivity was found for LCL161, showing higher activity in PDOs from the second and third resections, with 5.8 and 4.4-fold higher _GR_DSS compared to T1 PDOs, respectively, and far lower IC_50_ than the maximum plasma concentration of LCL161 [36] (Fig. 3A). The molecular hallmark of LCL161 activity is degradation of the targets c-IAP1, c-IAP2 and XIAP [19, 36, 37], and this was evident in post-treatment PDOs of all five lesions (Fig. 3B). Protein expression of XIAP and Survivin was highest in the resistant T1 PDOs, both after treatment with DMSO (control) and LCL161. In contrast, c-IAP1, c-IAP2 and XIAP were almost completely degraded after LCL161 treatment in the sensitive T2-T5 PDOs (Fig. 3B). Furthermore, treatment with LCL161 resulted in induction of apoptosis in a concentration- and time-dependent manner in the sensitive T3 PDOs, but not in the T1 PDOs, as indicated by increased PARP-, Caspase 7-, Caspase 8 and Caspase 3 activation (Fig. 3C).

Important mediators of TNF alpha signaling such as *TNF, TNFRSF12A*, three *RIPK* gene family members and genes encoding other apoptosis regulators such as *CASP9, BAK1* and *BIRC3* had expression levels positively correlated with LCL161 sensitivity across the T1-T5 and reference PDO dataset collectively (Fig. 3D). Strongest correlation with LCL161 activity was found for *RIPK1 (*Pearson r = 0.6, p < 0.0001*),* which also showed significant correlation among the T1-T5 PDOs separately (Pearson r = 0.92, p = 0.03). Furthermore, GSVA scores of the “Hallmark” gene set collection showed enrichment with the “TNFA signaling via NFKB” signature in all five PDOs from this patient, compared to the reference PDO dataset (Fig. 3E). This signature was also significantly associated with the gene sets of epithelial-mesenchymal transition, TGFβ, upregulated KRAS, inflammation, and apoptosis, both in liver metastasis tissue samples [9] and corresponding PDOs, suggesting molecular interactions of the pathways (Fig. 3F). Notably, LCL161 sensitivity in PDOs was significantly correlated with GSVA scores of almost all of these gene sets (Fig. 3E), and most strongly with inflammatory response (Parsons’s r = 0.44, p = 0.0012).

## Discussion

Stratification of cancer patients according to molecular and functional tumor characteristics can improve treatment outcomes [17]. Here, we employed a multidisciplinary approach to analyze spatio-temporal pharmacogenomic heterogeneity in a patient with recurrent, *KRAS* mutated liver metastases from rectal cancer. Ex vivo co-clinical analyses of standard, neoadjuvant combination chemotherapies at three consecutive liver resections modeled the dynamics of clinical treatment responses, including indications of acquired resistance to FLOX. Furthermore, a switch in systemic treatment from FLOX to FLIRI corresponded to a higher e*x vivo* activity of oxaliplatin, indicating re-sensitization.

*KRAS* mutation in a microsatellite stable background and an undifferentiated histopathology are parameters associated with an unfavorable patient prognosis and limited systemic treatment options after development of resistance to standard therapies [18]. This case report indicated vulnerability to the experimental SMAC mimetic LCL161, showing higher drug activity in the recurrent liver metastases. LCL161 sensitivity patterns were supported by mechanistic analyses of the drug targets and TNF-α signaling, indicating target engagement and a potential for response prediction by *RIPK1* gene expression levels, consistent with previously published clinical data in breast cancer [38]. Furthermore, IAP inhibitors can induce anti-tumor immunity [39, 40], and the strong inflammatory response in T1-T5 PDOs supports potency of a combination treatment of LCL161 and immune modulatory drugs. Combination with anti-PD1 therapy is currently being tested in a phase I study in patients with CRC (ClinicalTrials.gov Identifier: NCT02890069).

In conclusion, this case report supports that ex vivo pharmacotranscriptomics can model longitudinal treatment efficacy in metastatic CRC. It also supports further investigation of LCL161 as an anticancer agent, although clinical translation of results from this case report was not possible due to the lack of ongoing studies of IAP inhibitors in Norway.

## Data Availability

All datasets analyzed during the current study are available from the corresponding author on reasonable request.

## Abbreviations

5-FU: 5-Fluorouracil
CDX2: Caudal type homeobox 2
CK20: Cytokeratin 20
CK7: Cytokeratin 7
CRC: Colorectal cancer
ECAD: E-cadherin
FLIRI: 5-Fluorouracil, leucovorin, and irinotecan
FLOX: 5-Fluorouracil, leucovorin, and oxaliplatin,
FLV: 5-Fluorouracil and leucovorin,
_GR_DSS: Growth adjusted drug sensitivity scores
GSVA: Gene set variation analysis
IAP: Inhibitor of apoptosis protein
MRI: Magnetic resonance imaging
NF-κB: Nuclear factor kappa-B
PDO: Patient-derived organoid
RIPK1: Receptor-interacting protein kinase 1
SMAC: Second mitochondria-derived activator of caspases
TNF: Tumor necrosis factor
